# AI Virtual Human–Augmented Game-Based Teaching to Enhance Emotional Intelligence in Nursing Students: Protocol for a Single-Group Pretest-Posttest Action Research Study

**DOI:** 10.2196/80290

**Published:** 2025-10-17

**Authors:** Yung-Chieh Ching, Yen-Chung Ho

**Affiliations:** 1 Department of Nursing College of Nursing Asia University Taichung Taiwan

**Keywords:** emotional intelligence, artificial intelligence, AI virtual patient, game-based learning, nursing education innovation, protocol

## Abstract

**Background:**

The rapid advancement of IT and the complexity of health care demand innovative nursing education that moves beyond lectures and workshops. Nursing students must acquire clinical knowledge alongside emotional intelligence (EI), empathic communication, and crisis management to respond to patients at risk for suicide. In Taiwan, suicide is the second leading cause of death among university students, underscoring the urgency of suicide prevention training. Generative artificial intelligence (AI) platforms such as AI virtual humans provide immersive, scenario-based simulations that can integrate game-based learning into psychiatric nursing curricula.

**Objective:**

This study evaluates the effectiveness of combining game-based pedagogy with AI virtual human simulations in enhancing nursing students’ EI, empathic communication, and psychological crisis management. Specifically, it aims to (1) foster self-awareness and emotion regulation, (2) strengthen crisis assessment and coping strategies, (3) increase motivation and engagement, and (4) establish an evidence-based framework for nursing education innovation.

**Methods:**

An action research design will be conducted over 2 instructional cycles (36 weeks) within a psychiatric nursing course at a Taiwanese university. Participants are third-year postbaccalaureate nursing students recruited through convenience sampling. In phase 1, five simulation scenarios involving suicide risk presentations—major depressive disorder, bipolar disorder, schizophrenia, substance use disorder, and borderline personality disorder—were developed and validated by experts. Phase 2 applies 2 action research cycles (week 1 to 18 and week 19 to 36) with a single-group pretest-posttest design. Quantitative data include the Adult Emotional Intelligence Scale, administered at baseline and after the intervention. Qualitative data consist of reflection journals and classroom observations. Analysis employs descriptive statistics, paired sample 2-tailed *t* tests or Wilcoxon signed-rank tests, effect sizes, linear mixed-effects models, and thematic analysis.

**Results:**

Scenario development and expert review were completed in phase 1. Cycle 1 (weeks 1-18) has begun from September 2025, with 37 students enrolled, exceeding the sample size target of 32. Postintervention Adult Emotional Intelligence Scale results will be collected by week 16, followed by cycle 2 with a new cohort. Final integration and analysis will occur after week 36 to evaluate intervention effectiveness.

**Conclusions:**

Integrating game-based learning with AI virtual human simulations introduces an innovative, technology-enhanced model for psychiatric nursing education. Anticipated outcomes include improved EI, enhanced empathic communication, and stronger crisis management abilities. Through iterative refinement across 2 cycles, this study will provide empirical evidence on the role of generative AI in nursing education, informing curriculum innovation and supporting AI-driven simulation as a sustainable strategy for competency-based training.

**International Registered Report Identifier (IRRID):**

PRR1-10.2196/80290

## Introduction

### Background

From the perspective of nursing education, the rapid advancement of IT and the continuous evolution of medical knowledge have significantly impacted health care delivery models and the demand for clinical competencies. Modern nurses are expected not only to possess fundamental clinical skills but also to demonstrate professional knowledge, problem-solving ability, critical thinking, and effective collaboration within interdisciplinary teams. Traditional teaching approaches such as lectures, demonstrations, seminars, and workshops are increasingly insufficient to meet the needs of student-centered learning models. A recent meta-analysis found that virtual simulation significantly enhanced clinical reasoning skills among nursing students, especially when scenarios were longer than 30 minutes as well as nonimmersive and followed by debriefing [1]. In response, nursing education has gradually shifted toward innovative and diversified teaching strategies, including problem-based learning, clinical reasoning, and scenario-based simulation. These approaches aim to foster self-directed learning, integrate theory into clinical practice, enhance motivation, promote peer discussion, and cultivate lifelong learning skills.

Generative artificial intelligence (AI), including tools such as ChatGPT and virtual assistants, has been introduced into educational contexts to support personalized learning, prepare students for complex clinical situations, and improve engagement. A scoping review [2] categorized AI-based teaching strategies in nursing into simulation-based learning, AI-augmented instruction, and AI-generated content and tools and reported improvements in clinical understanding and student participation. In Taiwan, research on AI literacy among nursing students, such as the integration of ChatGPT and Copilot into academic writing courses, has shown significant gains in AI competence and writing performance [3].

From a public health perspective, suicide remains a critical global issue. According to the World Health Organization [4], more than 700,000 people died by suicide in 2019—more than from HIV, malaria, breast cancer, or war—making it one of the leading causes of death worldwide. World Health Organization data indicate that 1 in every 100 deaths is due to suicide, prompting new global policies to strengthen suicide prevention efforts and improve training for health care professionals.

In Taiwan, suicide rates have risen steadily in recent years, particularly among individuals aged 15 to 24 years. According to the Ministry of Health and Welfare [5], suicide is the second leading cause of death among young people, with university students facing heightened risks related to academic pressure, interpersonal stress, and mental health challenges. A series of student suicides in 2020 drew national attention to the complexity of suicide-related factors such as academic stress, emotional distress, and interpersonal difficulties. These incidents have highlighted the need for improved mental health awareness and support within educational settings, enabling both students and faculty to better recognize psychological distress, regulate emotions, and manage stress effectively.

International studies suggest that short-term suicide prevention education can enhance nursing students’ confidence, skills, and attitudes toward suicide prevention. However, suicide prevention programs in most Taiwanese universities remain limited to optional “gatekeeper” lectures or brief workshops, making it difficult to evaluate their effectiveness or ensure knowledge transfer to clinical practice. Given that frontline nurses frequently encounter patients at risk for suicide—including those outside of psychiatric departments—there is a pressing need to integrate effective suicide assessment training into nursing curricula. This forms the foundation of this teaching research project, which aims to use generative AI to create AI virtual human simulations. These simulations will allow students to apply knowledge learned in the classroom by engaging in virtual assessment conversations with AI virtual patients, thereby enhancing the students’ suicide risk assessment and recognition skills.

Nursing students not only face academic demands but also the psychological pressures of clinical internships, such as managing emotional responses during patient and family interactions or witnessing medical emergencies. These stressors can lead to emotional distress, anxiety, or burnout. Despite growing awareness of these challenges, few interventions have systematically addressed the dual need for emotional intelligence (EI) development and suicide prevention training in Taiwanese nursing education. Therefore, this study proposes an innovative AI-based teaching approach that integrates EI enhancement, empathic communication, and crisis management skills through game-based learning (GBL) and AI virtual human simulations. The specific aim of this study is to evaluate the effectiveness of this integrated model in improving nursing students’ EI, suicide risk assessment, and psychological crisis management skills, thereby addressing a critical gap in current literature.

### EI Among University Students and Its Relevance in Nursing Education

EI has become a significant focus in mental health research among university students in Taiwan, particularly due to its association with reduced psychological distress and enhanced coping abilities. Multiple studies have shown that higher EI is positively correlated with better mental health outcomes, including lower levels of stress and anxiety; for instance, a study of university students in southern Taiwan revealed a positive relationship between EI and interpersonal relationships, with female students scoring higher in EI than their male counterparts [6]. Another study identified EI as a protective factor that helps buffer the negative psychological effects of stress and adverse life events [7]. In addition, EI can be enhanced through education. A study evaluating an emotion regulation course for Taiwanese university students reported significant improvements in EI scores and reductions in psychological distress after the intervention, highlighting the effectiveness of education-based approaches to fostering EI [8].

In the context of nursing education, EI is not only important but also foundational. The development of self-awareness is a key starting point, as it enhances communication, clinical decision-making, and interpersonal competence. Research has demonstrated a positive correlation between self-awareness and communication ability among nursing students, and courses that emphasize self-awareness have been shown to significantly improve these skills [9]. Furthermore, the integration of mindfulness with self-awareness has proven effective in helping nursing students manage emotional challenges such as countertransference, thereby improving emotion regulation and professional behavior [10].

Emotion awareness and EI are particularly critical in psychiatric nursing, where students must respond to patients with emotionally complex needs. A study by Por et al [11] found that higher levels of EI were significantly associated with better stress management, clinical decision-making, and subjective well-being among nursing students. However, more recent research has revealed that while nursing students may perform relatively well in managing social emotions, they often score lower in emotion awareness—indicating a gap that may hinder emotion regulation in clinical settings. As such, it has been recommended that EI training be formally integrated into nursing curricula to strengthen critical thinking and decision-making skills, leading to improved educational and clinical outcomes [12].

### Integration of GBL Into Nursing Education

GBL has emerged as an innovative educational strategy that incorporates game design elements to enhance engagement, enjoyment, and learning outcomes. While nursing education traditionally emphasizes the development of professional skills and critical thinking, conventional instructional methods often fall short in motivating students or sustaining their interest. As a result, a growing body of research has examined the effectiveness of applying GBL within nursing curricula.

Studies have demonstrated that GBL contributes to improved student motivation and academic performance; for instance, a systematic review by Xu et al [13] found that GBL significantly enhanced nursing students’ learning experiences, particularly by increasing motivation and emotional engagement. Similarly, Reed reported that game-based teaching approaches not only improved knowledge retention [14] and test performance but also elicited more positive student feedback and engagement during class.

A randomized controlled trial by Blanié et al [15] compared game-based simulation with traditional teaching for developing clinical reasoning skills among second-year nursing students in France. Participants were randomly assigned to either a group that used interactive game-based case simulations or a group that studied the same material through text-based methods. Both groups were evaluated using the script concordance test immediately after instruction and again 1 month later. While no significant differences were found between the 2 groups in immediate or delayed clinical reasoning performance, the study highlighted higher levels of student satisfaction and learning motivation in the simulation by gaming game-based simulation group. Students reported that the game simulations provided a more engaging, interactive, and contextually meaningful learning experience.

These findings suggest that GBL holds substantial educational potential in nursing education, particularly in enhancing student motivation, satisfaction, and selected clinical competencies. However, successful implementation requires careful consideration of design, technological integration, and contextual factors. Further research is needed to explore the long-term effects of GBL across diverse educational settings and nursing specialties.

### Integration of AI Technology Into Nursing Education

With the rapid advancement of IT, nursing education has increasingly incorporated various forms of digital and mobile learning interventions. These include mobile apps, electronic learning platforms, and immersive technologies such as augmented reality and virtual reality (VR). In recent years, the global rise of AI has further expanded educational possibilities, although AI has been more extensively developed and applied in clinical health care practice than in nursing education. The emergence of generative AI technologies—particularly after the release of ChatGPT in November 2022—has signaled a transformative shift in education. In light of these developments, nursing education must adapt accordingly.

A meta-analysis by Chen et al [16] on mobile learning interventions in nursing education revealed that, compared to traditional lecture-based instruction, mobile learning strategies significantly improved students’ clinical knowledge, nursing skills, and confidence, while also receiving higher satisfaction ratings from students. In a recent quasi-experimental randomized controlled trial involving 166 third-year nursing students, the effectiveness of simulated learning using AI virtual patients was compared to that of standardized patient simulations. Both methods were found to enhance clinical safety awareness, reduce errors, and improve clinical competence and decision-making. However, students in the AI virtual patient group demonstrated significantly better outcomes in anxiety reduction, confidence building, and decision-making performance, with results reaching statistical significance (*P*<.001). These findings suggest that AI-based simulation in nursing education not only supports the acquisition of evidence-based clinical knowledge but also enhances communication, critical thinking, and patient safety skills [17].

Although nursing has long intersected with technology, the development and application of AI have typically been seen as the domain of computer science, software engineering, and robotics. However, educators should no longer rely solely on traditional classroom methods. Instead, they must actively enhance their technological competencies by integrating tools such as ChatGPT to support content creation and instructional design. When combined with clinical expertise, generative AI technologies can lead to the rapid development of innovative, high-quality teaching materials that increase student engagement and learning outcomes.

In this project, faculty members have been granted exclusive authorization to use the Virti AI virtual human scenario design platform, developed by HTC. After receiving training on AI-generated dialogues using ChatGPT, educators can create customized virtual patient simulations in as little as 30 minutes—enhancing efficiency by a factor of 10 to 100. Virti’s integration of natural language processing further enables dynamic, student-led interactions with AI-simulated patients, promoting clinical reasoning, communication, and knowledge retention. This AI-powered educational approach has the potential to transform nurse training by significantly improving humanistic communication skills in patient care settings.

### Aim

This study has 4 main objectives. First, it aims to enhance nursing students’ EI by helping them better recognize and understand their own emotional responses and those of others, using reflective and interactive simulations. Second, it seeks to strengthen students’ crisis management abilities through virtual patient scenarios that simulate high-stress situations, allowing them to practice appropriate coping and decision-making strategies. Third, the project aims to improve student engagement and motivation by integrating game-based and AI-enhanced learning, assessing their impact on participation, interest, and satisfaction. Finally, the study intends to provide empirical support for innovative nursing education by establishing a framework that combines game-based pedagogy with AI simulation to meet the evolving demands of clinical practice. This approach integrates practice and research through iterative cycles of planning, action, observation, and reflection to address real-world instructional challenges and support continuous improvement.

## Methods

### Overview

The innovative teaching strategy in this project integrates traditional classroom lectures, clinical case discussions, and instructional video analyses with additional components such as GBL, hands-on technical practice, and interactive software using AI virtual humans. These combined approaches aim to support nursing students in developing EI—particularly self-awareness and emotion recognition regulation—along with empathic communication skills. Furthermore, students will engage in suicide risk assessment training with virtual patients representing various psychiatric conditions.

**Figure 1:**
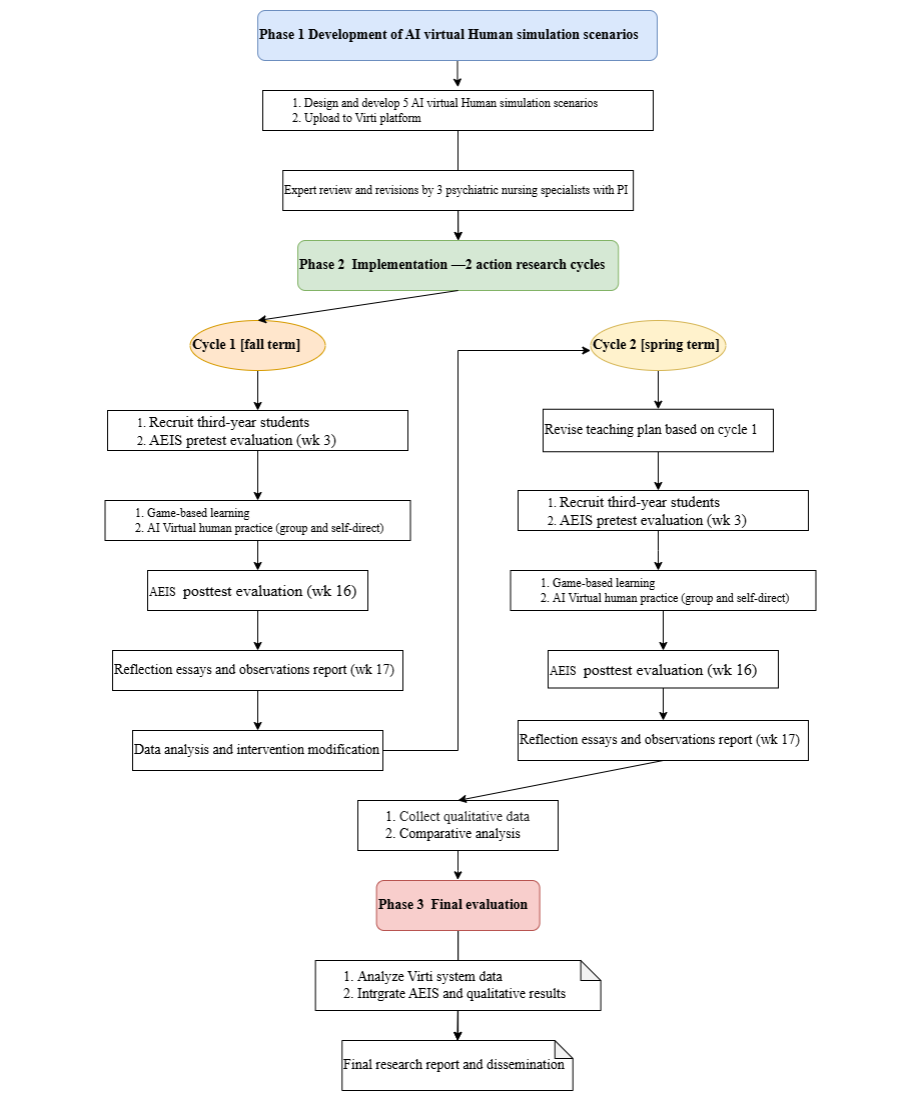
Research flowchart. AEIS: Adult Emotional Intelligence Scale; PI: principal investigator.

This teaching research project aims to explore how the integration of game-based pedagogy and AI virtual human interaction can enhance nursing students’ EI and psychological crisis management abilities. To achieve this, the study will use an action research methodology involving 2 iterative cycles. After implementing the first cycle and collecting both quantitative and qualitative data, revisions will be made to the intervention based on the findings before initiating the second cycle. Each cycle will incorporate a single-group pretest-posttest design to evaluate the effectiveness of the intervention using quantitative measures. Through this iterative process, the study seeks to optimize the impact of innovative teaching methods on students’ learning outcomes. The research design flowchart is shown in Figure 1.

### Phase 1: Development of an Innovative Teaching Plan Incorporating Generative AI Virtual Humans

During this phase, generative AI technologies will be applied to develop 5 AI virtual human simulation scenarios, which will be hosted on the platform provided by HTC. These include 1 scenario focused on empathic communication skills and comprehensive assessment of 5 core psychiatric domains, those scenarios representing different psychiatric diagnoses or clinical conditions: major depressive disorder, bipolar disorder (depressive episode), schizophrenia, substance use disorder, and borderline personality disorder. Each scenario will feature AI-simulated patients exhibiting suicide ideation and behaviors associated with suicide risk.

The principal investigator has obtained certification as a seed instructor for immersive VR teaching scenario development through the HTC medical VR program (Virti VR platform). Accordingly, the project will use the Virti AI virtual human platform to design intelligent, interactive case scenarios using generative AI. The content of the 5 scenarios will undergo expert panel review by 3 psychiatric mental health nursing professionals and the principal investigator. These expert consultations will guide revisions to ensure that the simulation scenarios accurately reflect realistic communication patterns and clinical presentations found in psychiatric settings.

Once the AI virtual human scenarios are finalized, students will be able to access them anytime and anywhere during phase 2 via the web-based platform. They will engage in interactive simulations with virtual psychiatric patients, practicing empathic communication, emotion recognition, and skills in psychological crisis and suicide risk assessment. All student-AI interactions will be automatically recorded on the backend of the system to facilitate evaluation and scoring by instructors.

### Phase 2: Implementation Phase—Integrating GBL and Generative AI Virtual Human Instruction (2 Action Research Cycles With Pretest-Posttest Evaluation in Each Cycle)

#### Design

This teaching research project adopts an action research methodology, which is a practice-oriented and participatory approach that emphasizes solving real-world problems through iterative cycles of planning, action, observation, and reflection. Action research is widely applied in diverse fields, including education, health care, management, and the social sciences. At its core, it involves close collaboration between researchers and participants to implement systematic actions and evaluations that lead to both practical improvements and theoretical development [18]. In recent years, action research has been increasingly recognized as a “multimethodology” approach due to its flexibility in integrating both qualitative and quantitative methods to address complex problems [19]. The typical process of action research consists of five key steps: (1) identifying the problem and defining the research objectives; (2) planning the action, including designing the intervention and anticipating possible outcomes; (3) implementing the action and collecting relevant data; (4) evaluating the outcomes to determine whether the objectives have been achieved; and (5) reflecting on and refining the action plan for subsequent cycles. The strengths of action research lie in its practical orientation and its participatory nature, which fosters the growth of knowledge and skills among participants [20].

EI refers to the ability to perceive, understand, and manage one’s own emotions as well as those of others. Its core components include self-awareness, self-regulation, social awareness, and relationship management [21]. In recent years, EI has gained widespread attention in both educational and clinical psychology domains. In Taiwan, researchers have developed a culturally adapted Adult Emotional Intelligence Scale (AEIS) [22], which has demonstrated strong reliability and validity in both nursing professionals and university student populations. Higher total scores on the AEIS indicate greater EI [22]. Accordingly, this teaching research project will be conducted through 2 iterative action research cycles. A single-group pretest-posttest design will be used for the quantitative component, while qualitative data will be collected through student reflective writings and classroom observations to analyze their emotion awareness, communication behaviors, and responses to the teaching intervention.

#### Sampling and Recruitment

Participants will be recruited from third-year nursing students enrolled in the Mental Health and Psychiatric Nursing course at a university in Taiwan. Convenience sampling will be used. The inclusion criteria are as follows: (1) students must be aged at least 20 years; and (2) they must be currently enrolled in the course, voluntarily agree to participate in the study, and be willing to complete both the pretest and posttest AEIS questionnaires as well as submit a written reflection. Students who have suspended their studies or are on academic leave at the time of recruitment will be excluded. Recruitment will be conducted through in-class announcements and information sheets provided by the course instructor. All participants will receive detailed explanations about the study procedures and their rights, and written informed consent will be obtained before participation.

#### Data Collection and Analysis Plan

This study adopts a single-group pretest-posttest design implemented across 2 instructional cycles (first and second semesters). Quantitative data will be gathered with the AEIS—a validated 26-item instrument covering 4 subscales: emotion awareness (items 1-7), emotional expression (items 8-12), emotion regulation (items 13-17), and emotional use (items 18-26). Each item is scored on a 4-point Likert scale (ranging from 0=strongly disagree to 3=strongly agree); higher scores denote greater EI [22]. Permission to use the AEIS has been obtained from the original author. The scale will be administered in week 3 (pretest evaluation) and week 16 (posttest evaluation) of each semester. Qualitative data will consist of students’ reflective reports—collected throughout both cycles—and classroom observations conducted in week 17 to document behavioral engagement during gameplay and AI-patient interactions.

At the item level, AEIS subscale scores will be prorated when no more than 1 item per subscale is missing; otherwise, the subscale will be set to missing. At the record level, patterns of missingness will be inspected with the Little MCAR test [23]. As linear mixed effects models (LMMs) provide unbiased estimates under a missing-at-random assumption, incomplete cases will be retained in the primary analyses. For ancillary paired sample 2-tailed *t* tests or Wilcoxon signed-rank tests, missing posttest values will be multiply imputed (20 imputations using predictive mean matching); the imputed results will be pooled following the rules formulated by Rubin [23] and compared with complete-case outputs as a sensitivity check.

Descriptive statistics (means and SDs or medians and IQRs) will be reported for all variables. The normality of pretest-posttest difference scores will be checked with the Shapiro-Wilk test. If the distribution is normal, within-participant changes in AEIS total and subscale scores will be analyzed with paired sample 2-tailed *t* tests; otherwise, the Wilcoxon signed rank test will be used. Effect sizes will be expressed as Cohen *d* with 95% CIs. To compare change trajectories between cycles, an LMM with random intercepts for participants will be fitted, including fixed effects for time (before the intervention vs after the intervention), cycle (1 vs 2), and their interaction (time × cycle). Holm-Bonferroni adjustment will control the family-wise error rate across AEIS subscales. All analyses will follow an intention-to-treat principle (participants with ≥1 posttest record retained); sensitivity checks with complete-case data will evaluate robustness. Statistical significance is set at α=.05 (2-tailed). Analyses will be conducted in SPSS software (version 29.0; IBM Corp) and R (version 4.4.0; R Foundation for Statistical Computing; *lme4* package for LMMs).

To capture students’ experiences and perspectives, we collected qualitative data through structured reflective reports and oral and written feedback after the implementation of the innovative teaching practices. Each student was required to complete a reflective report structured around 5 open-ended questions aligned with the core learning domains of self-awareness, emotional self-awareness, social awareness, empathic communication, and crisis management competence; for example, students described personal insights gained from participating in the card-based educational game, emotional reactions experienced during gameplay, and strategies for recognizing and responding to peers’ emotional cues. They also reflected on their ability to demonstrate empathy in simulated dialogues with AI virtual patients and articulated how these experiences contributed to their confidence in managing psychological crises. Each reflective entry was expected to exceed 100 Chinese characters, thereby encouraging deeper engagement and detailed narrative accounts of their learning processes.

In addition to written reflections, student oral and written feedback were gathered during debriefing sessions immediately after the teaching interventions. These interactive discussions invited participants to share their impressions of the AI virtual patient encounters and the gamified learning with card play, highlighting both challenges and perceived benefits. Students frequently commented on the authenticity of the AI-driven dialogues, which prompted them to practice empathic responses, and on the playfulness of the card game, which fostered self-discovery and peer interaction. Oral and written feedback not only provided spontaneous, unstructured accounts but also allowed for triangulation with the reflective reports, offering complementary insights into how the innovative teaching strategies shaped EI and learning engagement. Together, these qualitative materials provided a rich and multifaceted dataset for thematic analysis, illuminating both individual developmental trajectories and collective learning dynamics within the cohort.

During the practical sessions with AI virtual patients, investigators used structured observation rubrics to evaluate student performance across five domains: (1) the ability to recognize the patient, introduce themselves, and demonstrate proactive caring; (2) the use of appropriate communication skills during interviews or technical procedures; (3) the demonstration of empathy in patient interactions; (4) the accuracy of relevant assessments such as symptom evaluation or suicide risk assessment; and (5) the quality of group reflective sharing after practice. Each domain was equally weighted at 20 points, for a total of 100 points in the rubric.

Student reflection reports and student oral and written feedback from the implementation of the innovative teaching practices and observation notes will be thematically analyzed in NVivo 14 (Lumivero) using the 6-step framework proposed by Braun and Clarke [24]. The analysis will proceed systematically through 6 phases: (1) familiarization with the data through repeated reading and note taking, (2) generating initial codes across the dataset, (3) searching for candidate themes by collating codes, (4) reviewing themes in relation to both coded extracts and the entire dataset, (5) defining and naming themes to refine their scope and focus, and (6) producing a coherent report with representative extracts. To ensure rigor and transparency, coding decisions will be documented in an analytic memo trail, and at least two researchers will independently code a subset of data before discussing discrepancies to enhance reflexivity and credibility. The analytic approach will follow recent guidance that emphasizes active, reflexive engagement by the researcher rather than striving for consensus coding or reliability metrics, thereby acknowledging the researcher’s interpretative role in meaning making [24]. In addition, Braun and Clarke [25] highlighted common pitfalls and provided 10 recommendations for producing methodologically coherent thematic analysis, underscoring the importance of distinguishing between topic summaries and interpretative meaning-based themes. Their commentary further emphasizes that researchers must “own” their perspectives, ensuring that analytic practices remain theoretically consistent with the chosen thematic analysis approach [25]. Qualitative findings will be triangulated with quantitative results to elucidate mechanisms underlying observed changes in EI and learning engagement.

#### Cycle 1: Sampling and Recruitment

This cycle will be implemented during the first semester (fall term) of the third year in the nursing program. An innovative course will be introduced, incorporating GBL strategies and hands-on practice with an AI virtual human platform. Each student will be provided with a personal log-in account to access the system. During the practical sessions, students will work in small groups to engage in guided online simulations. In addition, they will be encouraged to access the platform outside of class time to enhance their empathic communication, emotion recognition, suicide risk assessment, and clinical decision-making skills. Both quantitative and qualitative data will be collected during this phase to inform revisions and improvements for the second cycle of the action research.

#### Cycle 2: Sampling and Recruitment

This cycle will be conducted during the second semester (spring term) of the third year for a new cohort of nursing students. On the basis of the results, feedback, and reflective evaluations gathered from faculty and students during the first cycle, the instructional content and design of the GBL activities and AI virtual human interactions will be refined. The revised intervention will then be implemented as part of a second action research cycle, with the aim of enhancing the overall effectiveness of the innovative teaching approach and achieving the study’s educational objectives.

### Phase 3: Final Evaluation of the Innovative Teaching Intervention

At the conclusion of the 2-semester implementation period, the principal investigator will conduct a comprehensive evaluation of student learning outcomes. This will include analysis of system-generated learning data from the Virti platform, along with the pretest and posttest results from the AEIS administered during both cycles. A comparative analysis of students’ learning outcomes across the 2 implementations will be conducted, integrating both quantitative and qualitative data. The final phase will conclude with the preparation of a comprehensive research report summarizing the findings and implications of the teaching innovation.

**Figure 2:**
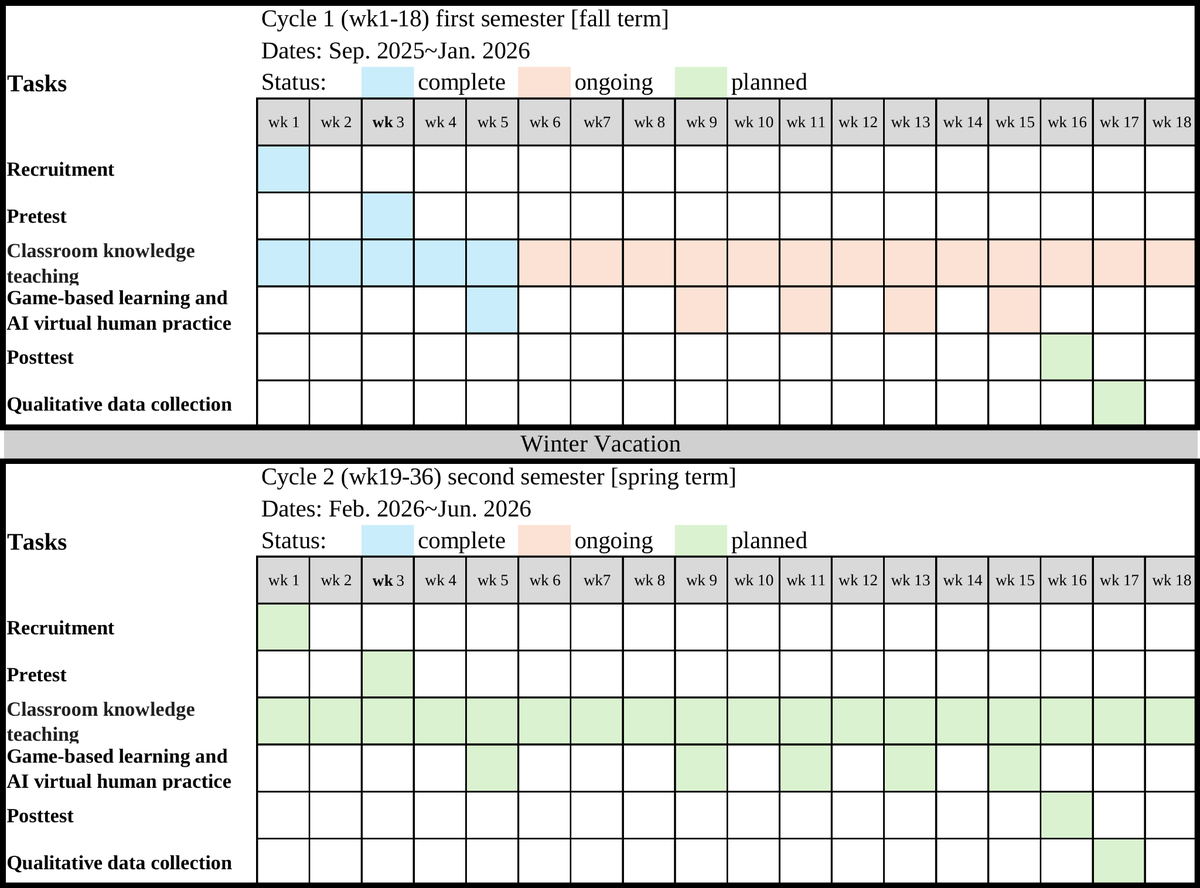
Combined SPIRIT (Standard Protocol Items: Recommendations for Interventional Trials) schedule of enrollment, interventions, and assessments for cycle 1 (weeks 1-18) and cycle 2 (weeks 19-36).

Figure 2 presents the combined SPIRIT (Standard Protocol Items: Recommendations for Interventional Trials) 2013 schedule of enrollment, interventions, and assessments for the full 36-week study. Consistent with the SPIRIT 2013 recommendations, the figure maps all key events across 2 action research cycles: cycle 1 (weeks 1-18) and cycle 2 (weeks 19-36). Shaded cells pinpoint the specific weeks in which each activity occurs: enrollment (week 1 and week 19), baseline administration of the AEIS (week 3 and 21), weekly game-based workshops, AI virtual human practice sessions, biweekly reflection logs (even-numbered weeks), and postintervention AEIS assessments (week 18 and week 36). A vertical dashed line at weeks 18-19 delineates the transition between cycles, providing a clear visual alignment of recruitment, intervention delivery, and outcome measurement throughout the study.

### Sample Size and Power Analysis

Using G*Power 3.1 (Heinrich Heine University) for a paired sample 2-tailed *t* test (“Means: difference between two dependent means, matched pairs”), we set α=.05, desired power=.80, and a medium anticipated effect size of Cohen *d*=0.50—consistent with meta-analytic estimates for short educational interventions targeting EI. The calculation yielded a required completer sample of 32 students to detect the pretest-posttest difference with 80% power. Allowing for typical nursing-course attrition of 15% to 20%, we plan to recruit 40 participants per cycle, ensuring that at least 32 remain for posttest analysis.

### Reporting-Guideline Compliance

This protocol has been prepared in accordance with the SPIRIT 2013 checklist, and the intervention is described in detail using the TIDieR (Template for Intervention Description and Replication) 12-item checklist. The completed SPIRIT and TIDieR checklists are provided as Multimedia Appendix 1 and Multimedia Appendix 2, respectively. As the study uses a single-group pretest-posttest design, we also plan to report outcomes following the TREND (Transparent Reporting of Evaluations with Nonrandomized Designs) 22-item statement; a draft TREND checklist will be submitted alongside the future results manuscript.

### Rigor in Qualitative Data

The qualitative component of this study, which includes students’ reflective reports, oral feedback, and structured observation notes, will be conducted with attention to methodological rigor. Following the criteria outlined by Lincoln et al [26], the study emphasizes transparency, reflexivity, and systematic documentation to ensure the trustworthiness of the findings.

Confirmability will be ensured through a coding record, allowing others to trace how findings were reached. The principal investigator, with years of psychiatric nursing and teaching experience, will keep reflexive journals to record assumptions and reflections. Triangulation across reflective reports, oral feedback, and observation notes will enhance confirmability, while peer debriefing with nursing experts will provide external checks for the AI virtual human scenario for patients with suicidal thoughts and mental illness. These strategies ensure that interpretations are grounded in data rather than influenced by researcher bias [26].

Dependability will be addressed by documenting each step of the research process, including scenario design, recruitment, data collection, and analysis. Iterative action research cycles provide opportunities to refine procedures while maintaining stability. Expert meetings will review progress and adapt interventions consistently. A methodological log and external expert audit will support transparency and replicability. Following the framework proposed by Lincoln et al [26], these practices demonstrate that the process is logical, traceable, and dependable.

Credibility will be supported through triangulation of multiple data sources and member reflections during debriefings. The principal investigator’s expertise in psychiatric nursing ensures prolonged engagement and contextual understanding. The methodological process will document how interpretations are shaped, while peer debriefing with a faculty member offers external validation. These approaches confirm that the findings authentically represent participants’ experiences, aligning with the standard for credibility in qualitative research established by Lincoln et al [26].

Transferability will be enhanced by providing thick descriptions of participants, course context, and intervention details, including AI virtual human simulations and game-based activities. Rich excerpts from student reflections and oral feedback will allow readers to judge the relevance of the findings for their own contexts. By situating the study within Taiwanese nursing education while highlighting the universal themes of EI and crisis training, the research facilitates applicability beyond its immediate setting [26].

### Ethical Considerations

This teaching project has been approved for funding (see Multimedia Appendix 3) and is under review by the central regional research ethics committee of China Medical University, Taichung, Taiwan review board (T-AU-36938) to ensure adherence to ethical standards in research involving human participants. All data collected in this study will be handled in accordance with institutional review board ethical guidelines to ensure participant privacy and data security. Participants will be assigned unique ID codes, and no personally identifiable information will be linked to the analysis. Digital data, including survey responses, reflection journals, and system use logs, will be stored on encrypted and password-protected institutional servers accessible only to the principal investigator and authorized research team members. All documents will be kept in locked cabinets in secure university offices. All data will be retained for a maximum of 5 years and then permanently destroyed in accordance with institutional policies. Results will be reported in aggregate form to prevent individual identification. Participation in this study is voluntary, and informed consent will be obtained from all participants before data collection. Participants may withdraw at any time without penalty or impact on their academic standing.

## Results

This project aims to develop 5 AI virtual human simulation scenarios. One scenario will focus on practicing empathic communication skills and conducting comprehensive psychiatric assessments across 5 core domains. The scenarios will present AI-simulated patients demonstrating suicide ideation and suicide risk related to different psychiatric diagnoses, including major depressive disorder, bipolar disorder (depressive episode), schizophrenia, and borderline personality disorder. The anticipated outcomes of this study include enhanced EI and improved psychological crisis management skills among nursing students. Furthermore, the findings are expected to contribute to an evidence-based foundation for innovative instructional strategies in nursing education that incorporate AI-driven simulation technology.

In terms of the research timeline, data collection will be conducted during cycle 1, including both qualitative and quantitative data. A preliminary analysis will be performed by the end of cycle 1, followed by a second expert panel meeting during the winter vacation. Based on the results of cycle 1 and expert feedback, the AI simulation lesson plans will be refined before the implementation of cycle 2. During cycle 2, both qualitative and quantitative data will again be collected. Upon completion of the 2 action research cycles, an overall data analysis will be conducted, and the final results and manuscript are expected to be completed and submitted within 3 months the research project is completed.

## Discussion

### Anticipated Findings

This project aims to comprehensively enhance the professional competencies and psychological resilience of third-year postbaccalaureate nursing students, preparing them for the challenges of future clinical practice. By integrating GBL with an AI virtual human platform, the intervention is designed to strengthen students’ EI, including their ability to recognize, understand, and manage emotions effectively. The program also seeks to improve students’ psychological crisis management skills, fostering adaptive coping and decision-making under stress. In addition, it aims to promote empathic communication and emotional connection with patients, while enhancing clinical judgment and problem-solving abilities through immersive, scenario-based learning. The innovative teaching strategies are expected to increase student motivation and engagement, making the learning process more interactive and appealing. Furthermore, the intervention will familiarize students with the application of AI technologies in nursing education, thereby improving their technological adaptability. Finally, the program encourages self-reflection and self-assessment to support ongoing professional development and lifelong learning.

### Strengths and Limitations

This study is expected to introduce an innovative educational approach by integrating GBL with AI virtual human interactions to enhance nursing students’ EI and psychological crisis management skills. A key anticipated strength lies in the use of experiential and technology-enhanced learning strategies within an action research framework, which may facilitate iterative refinement of teaching practices and encourage reflective learning. The planned use of both quantitative and qualitative methods is also likely to provide a comprehensive assessment of learning outcomes, capturing changes in knowledge, emotional competence, and communication behaviors.

Nevertheless, several potential limitations should be considered. As the study will adopt a single-group pretest-posttest design, the lack of a control group may limit the ability to draw causal inferences. However, implementing a control group in an educational setting may raise ethical concerns regarding unequal access to innovative teaching strategies, potentially leading to student dissatisfaction or perceptions of unfair treatment. In addition, the sample will be limited to third-year postbaccalaureate nursing students from a single institution, which may affect the generalizability of the findings. The AI virtual human platform, while offering interactive and standardized scenarios, may not fully capture the complexity and unpredictability of real-world clinical encounters. Furthermore, the relatively short duration of the intervention may limit the ability to evaluate sustained or long-term impacts on clinical competence and professional development.

### Conclusions

This study presents an innovative, technology-enhanced teaching model that integrates GBL and AI virtual human simulations into psychiatric nursing education. By combining traditional instruction with immersive and interactive elements, the project aims to enhance nursing students’ EI, empathic communication, and psychological crisis management skills. Through the implementation of 2 iterative action research cycles, the study is designed to evaluate and refine the intervention based on both quantitative outcomes from the AEIS and qualitative insights from student reflections and classroom observations. The use of generative AI technology and the Virti platform represents a novel approach to experiential learning in mental health nursing. The anticipated findings may offer valuable evidence to support the integration of AI-driven simulations into nursing curricula and provide a foundation for future innovations in competency-based education.

## Appendix

Multimedia Appendix 1 SPIRIT 2013 checklist.

Multimedia Appendix 2 TIDieR checklist.

Multimedia Appendix 3 The peer-review report from the Teaching Practice Research Project of the Ministry of Education, Republic of China (Taiwan).

## Abbreviations

AEIS: Adult Emotional Intelligence Scale
AI: artificial intelligence
EI: emotional intelligence
GBL: game-based learning
LMM: linear mixed effects model
SPIRIT: Standard Protocol Items: Recommendations for Interventional Trials
TIDieR: Template for Intervention Description and Replication
TREND: Transparent Reporting of Evaluations with Nonrandomized Designs
VR: virtual reality

## Data Availability

The datasets generated and analyzed during this study will be available from the corresponding author on reasonable request.
