# Supplementary material for: AI Virtual Human–Augmented Game-Based Teaching to Enhance Emotional Intelligence in Nursing Students: Protocol for a Single-Group Pretest-Posttest Action Research Study
Source: JMIR Res Protoc. 2025 Oct 17;14:e80290. doi: 10.2196/80290 (PMC12579293; doi:10.2196/80290)
Supplement: Multimedia Appendix 1 [file resprot_v14i1e80290_app1.docx]

# SPIRIT 2013 Checklist (Study Protocols of Interventional Studies)

|  | Item | Where addressed in protocol | Notes |
| --- | --- | --- | --- |
| 1 | Title | Title page | ✓ |
| 2 | Trial registration | Methods → Registration | OSF ID TBD |
| 3 | Protocol version / date | Title page footer | V1.0–2025‑07‑08 |
| 4 | Funding | Funding section | ✓ |
| 5 | Roles & responsibilities | Authors’ Contributions + Conflicts | ✓ |
| 6 | Background & rationale | Introduction | ✓ |
| 7 | Objectives | Aim subsection | ✓ |
| 8 | Trial design | Methods → Overview | ✓ |
| 9 | Study setting | Methods → Sampling & Recruitment | Classroom + Virti platform |
| 10 | Eligibility criteria | Methods → Sampling & Recruitment | Age ≥20, enrolled, consent |
| 11 | Interventions | Phase One & Two; see TIDieR appendix | ✓ |
| 12 | Outcomes | Methods → Data Collection & Analysis | Primary: AEIS total; Secondary: subscales |
| 13 | Participant timeline (figure) | Figure 1 | Timeline provided |
| 14 | Sample size | Methods → Sample Size | G*Power d=0.5 → 34 +20% attrition |
| 15 | Recruitment | Sampling & Recruitment | In‑class announcements |
| 18 | Data collection methods | Data Collection & Analysis | Surveys, logs, reflections |
| 19 | Data management | Methods → Data Security & Access | Encrypted server |
| 20 | Statistical methods | Statistical Analysis | Paired t / Wilcoxon; LMM |
| 24 | Research ethics approval | Ethical Considerations | IRB under review |
