# Supplementary material for: AI Virtual Human–Augmented Game-Based Teaching to Enhance Emotional Intelligence in Nursing Students: Protocol for a Single-Group Pretest-Posttest Action Research Study
Source: JMIR Res Protoc. 2025 Oct 17;14:e80290. doi: 10.2196/80290 (PMC12579293; doi:10.2196/80290)
Supplement: Multimedia Appendix 2 [file resprot_v14i1e80290_app2.docx]

TIDieR Checklist (Template for Intervention Description & Replication)

| # | Item | Information in protocol |
| --- | --- | --- |
| 1 | Brief name | AI Virtual Human–Augmented Game‑Based Teaching |
| 2 | Why (rationale) | Introduction → Background/Aim |
| 3 | Materials | Card game, Virti scripts, student devices |
| 4 | Procedures | Lecture → card game → AI simulation → debrief (weekly) |
| 5 | Who provided | PI + HTC‑trained assistants |
| 6 | How | Small‑group workshops + individual online practice |
| 7 | Where | Simulation classroom and personal devices |
| 8 | When & how much | 3 h/week class + ≥1 h self‑practice (18 weeks × 2) |
| 9 | Tailoring | AI responses adapt to student input |
| 10 | Modifications | Scripts refined between cycles |
| 11 | Planned fidelity | Virti logs; instructor checklist |
| 12 | Actual fidelity | Phase Three logfile analysis |
