# Supplementary material for: AI Virtual Human–Augmented Game-Based Teaching to Enhance Emotional Intelligence in Nursing Students: Protocol for a Single-Group Pretest-Posttest Action Research Study
Source: JMIR Res Protoc. 2025 Oct 17;14:e80290. doi: 10.2196/80290 (PMC12579293; doi:10.2196/80290)
Supplement: Multimedia Appendix 3 [file resprot_v14i1e80290_app3.pdf]

Review Results

Download plan information

Applicant Information | Basic information of the project | Course Plan | Research Project Content | Apply for Grants | Unaudited funding | Review Results | Plan Change History

Results of the review

Discipline/Project	[Project] Emotional Health and Well-being
Application period	One-year
Project Name	To explore the effectiveness of game-based teaching combined with AI virtual patient interactive learning program in promoting nursing students' emotional intelligence and psychological crisis management ability through action research method
Project No.	PEH1140261
Project Host	Jing Yongjie
Results of the review	pass
Review Opinion	The applicant has expertise in psychiatric nursing, rich course teaching experience and achievements, especially experience and certification in the implementation of digital courses, and the ability to design AI virtual patients. This application focuses on two aspects: "Mental health dilemmas and suicide issues among college students" and "Insufficient emotional intelligence and psychological crisis management training in nursing education". The project theme is clearly set and the course design is interesting. Through the combination of action research, game-based learning and AI virtual patients, in addition to enhancing nursing students' self-exploration, awareness of their own and others' emotions, and strengthening the development of emotional intelligence, the course is also expected to effectively reduce nursing students' depression and suicide risks. It is a research direction that is worth trying and exploring. In addition, the course evaluation is diverse, and the expected results and objectives are highly consistent. The overall plan has both theoretical and practical value. However, due to the rich course design and diverse evaluation methods, the actual implementation of the research is quite challenging, and the results of the plan need to be continuously tracked and observed.
Through the process	One-year period (2025-08-01 ~ 2026-07-31)
Subsidy	233,000 Yuan
Personnel Expenses (Doctoral Students) Subsidy Amount	This amount has not been applied for
Ethical review documents	Before implementing the plan, you need to submit a document showing that you have passed the research ethics review.

Back to the project list page
